# Molecular Mechanisms of Regulation and Action of microRNA-199a in Testicular Germ Cell Tumor and Glioblastomas

**DOI:** 10.1371/journal.pone.0083980

**Published:** 2013-12-31

**Authors:** Shen Gu, Hoi Hung Cheung, Tin Lap Lee, Gang Lu, Wai Sang Poon, Wai Yee Chan

**Affiliations:** 1 School of Biomedical Sciences, The Chinese University of Hong Kong, HKSAR, China; 2 Section on Clinical and Developmental Genomics, Eunice Kennedy Shriver National Institute of Child Health and Human Development, National Institutes of Health, Bethesda, Maryland, United States of America; 3 Department of Surgery, The Chinese University of Hong Kong, HKSAR, China; The Chinese University of Hong Kong, China

## Abstract

MicroRNA-199a (miRNA-199a) has been shown to have comprehensive functions and behave differently in different systems and diseases. It is encoded by two loci in the human genome, miR-199a-1 in chromosome 19 and miR-199a-2 in chromosome 1. Both loci give rise to the same miRNAs (miR-199a-5p and miR-199a-3p). The cause of the diverse action of the miRNA in different systems is not clear. However, it is likely due to different regulation of the two genomic loci and variable targets of the miRNA in different cells and tissues. Here we studied promoter methylation of miR-199a in testicular germ cell tumors (TGCTs) and glioblastomas (gliomas) and discovered that hypermethylation in TGCTs of both miR-199a-1 and -2 resulted in its reduced expression, while hypomethylation of miR-199a-2 but not -1 in gliomas may be related to its elevated expression. We also identified a common regulator, REST, which preferentially bound to the methylated promoters of both miR-199a-1 and miR-199a-2. The action of miR-199a is dependent on its downstream targets. We identified MAFB as a putative target of miRNA-199a-5p in TGCTs and confirmed that the tumor suppression activity of the microRNA is mediated by its target MAFB. By studying the mechanisms that control the expressions of miR-199a and its various downstream targets, we hope to use miR-199a as a model to understand the complexity of miRNA biology.

## Introduction

Ever since its discovery a decade ago[1], [2], it has been reported repeatedly that microRNA (miRNA) exhibited diverse trend of expression in different species and played crucial roles in most of the cellular processes[3]. Despite the large number of studies, the complicated and diversified biological roles of miRNAs are far from being understood.

miR-199a offers an excellent example to illustrate the complexity of the miRNA regulation and action [4]. After cleavage from its precursor and formation of the double stranded miRNA[5], [6], unlike most cases where the guide strand remains while the passenger strand is degraded, both strands from pre-miR-199a can form mature and functional miRNAs, namely miR-199a-3p and miR-199a-5p, respectively[7]. The two miRNAs have different sequences and recognize different seed regions and so different targets. In addition to its two mature forms, there are two loci that encode the precursor of miR-199a-3p and -5p in the human genome; one in Chromosome 1 (miR-199a-2 in Chr1, miRBase Accession MI0000281) and the other in Chromosome 19 (miR-199a-1 in Chr19, miRBase Accession MI0000242). As a result, there may be different regulatory mechanisms for the two chromosomes that control the expression of miR-199a under different circumstances, making the biological behaviors of miR-199a diverse and complicated.

miR-199a is well-conserved through different species[8], [9], and has been identified by diverse high-throughput screenings in many models and diseases. Expression of miR-199a could be down-regulated by epigenetic changes like DNA methylation[10], [11] and histone modification[12]. It could also be up-regulated by transcription factor activation (e.g. TWSIT1[13] and EGR1[14] binding on miR-199a-2's promoter in Chr1). The functions of miR-199a are quite complicated in different systems. For example, miR-199a is involved in cardiomyocytes protection by rapid down-regulation under hypoxic conditions and prompts HIF1a expression[15]. It is up-regulated and behaves as a major regulator during tissue fibrosis in lung fibroblasts[16] and livers[17]. In tumors, it could behave either as an oncogene (e.g. in gastric cancer[18], hepatoblastoma[19] and melanoma[20]), or as a tumor suppressor (e.g. in renal cell cancer[21], hepatocellular carcinoma[22], testicular germ cell tumors[23] and breast cancer[24]) in different cancer types or even in different cancer sub-types (e.g. up-regulation in ovarian cancer stem cells and down-regulation in serous ovarian cancer tissues[25], [26]).

Most studies of miRNAs only focus on a single system or disease model, which is neither thorough nor broad enough, especially for a sophisticated miRNA like miR-199a. In this report, we examined the dysregulation of miR-199a in two different types of tumors, testicular germ cell tumors (TGCTs) and glioblastomas (gliomas), and revealed its opposite expression patterns and different regulatory mechanisms. Since the functions of miRNAs could only be realized through its targets, we identified MAFB as a direct target under miR-199a-5p in TGCTs and explained the anti-proliferation effect of miR-199a-5p in TGCTs. This is the first time miR-199a was studied with a broader and more diverse approach in order to understand its regulation and function.

## Results

### Dysregulation of miR-199a in tumors were controlled by DNA methylation

**Figure 1:**
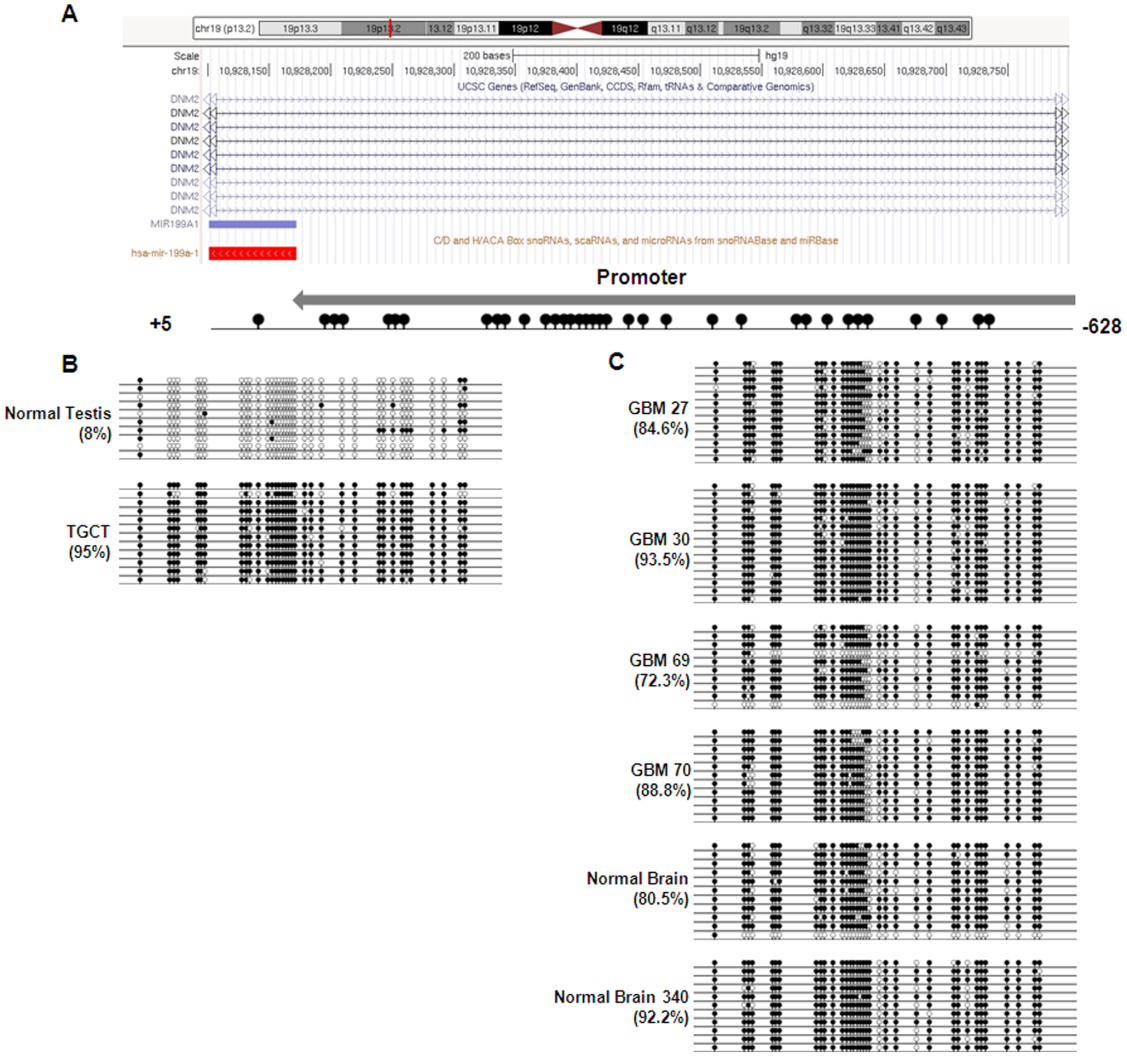
Methylation status of miR-199a-1 promoter in Chr19 in tumors. A). Genomic representation of differential methylation region from chr19:10,928,097-10,928,800 (hg19). Promoter of miR-199a-1 (+5:−628) embedded in intron-15 of DNM2 is indicated with relative locations of all CpG sites (lolipops). B). Hypermethylation (95%) of miR-199a-1 promoter was found in testicular germ cell tumor cells (NT2 cells) comparing to unmethylation in normal testis fibroblasts (HT cells). C). Hypermethylation of miR-199a-1 promoter was found in both normal brain DNA, Normal Brain 340 and glioma patient samples (GBM 27, 30, 69 and 70).

**Figure 2:**
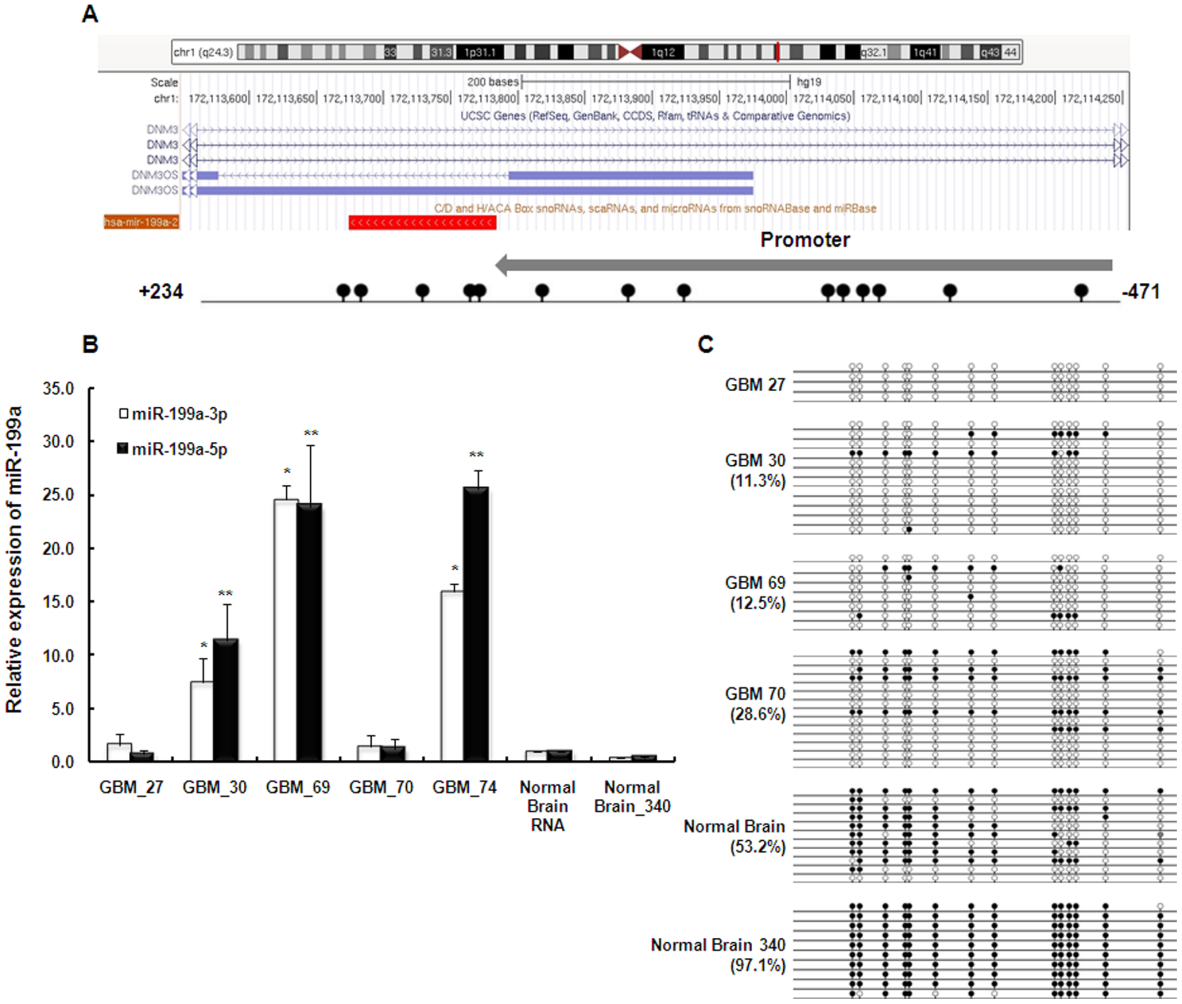
Elevated expression of miR-199a versus hypomethylation of miR-199a-2 promoter in Chr1 in glioma. A). Genomic representation of differential methylation region from chr1:172,113,550-172,114,255 (hg19). miR-199a-2 and its upstream promoter (+234:−471) embedded in intron-14 of *DNM3* is indicated with relative locations of all CpG sites (lolipops). B). qPCR showing increased expression of both miR-199a-3p and -5p in three out of five glioma patients compared with normal brain. *, p<0.05 miR-199a-3p expression of GBM samples comparing to normal brain; **, p<0.05 miR-199a-5p expression of GBM samples comparing to normal brain. C). Hypomethylation of miR-199a-2 was found in glioma patients (GBM 27, 30. 69 and 70) comparing to normal brain.

To examine the regulation of expression in both TGCTs and glioblastomas, the methylation status of the promoter of miR-199a in chromosome 1 (Chr1) (Figure 1A) and chromosome 19 (Chr19) (Figure 2A) was interrogated with bisulfite sequencing. Figure 1B showed that the promoter of miR-199a-1 in Chr19 was hypermethylated in NT2 cells comparing to HT cells (95% comparing to 8%). Positive methylation is defined as methylated CpG compared to unmethylated CpG in this study.

Among the gliomas patient samples examined, three out of five showed significantly elevated expression of both forms of mature miR-199a (miR-199a-3p and -5p) when compared to normal human brain (Figure 2B). Studies of methylation status revealed that promoter of miR-199a-1 in Chr19 was hypermethylated in all gliomas tissue samples and normal brain tissue while promoter of miR-199a-2 in Chr1 was less methylated in gliomas compared to normal brain (Figure 2A, 2C). Therefore, the increased expression of miR-199a in gliomas could be a consequence of hypomethylation of the promoter of the allele in Chr1. Because brain tissues, especially normal brain tissues, are very hard to come by, we assayed all the brain tissues we could manage to get, and the evidence did point to, at least within a subgroup of gliomas, a possible relationship between methylation and expression of miR-199a in the brain tissues

**Figure 3:**
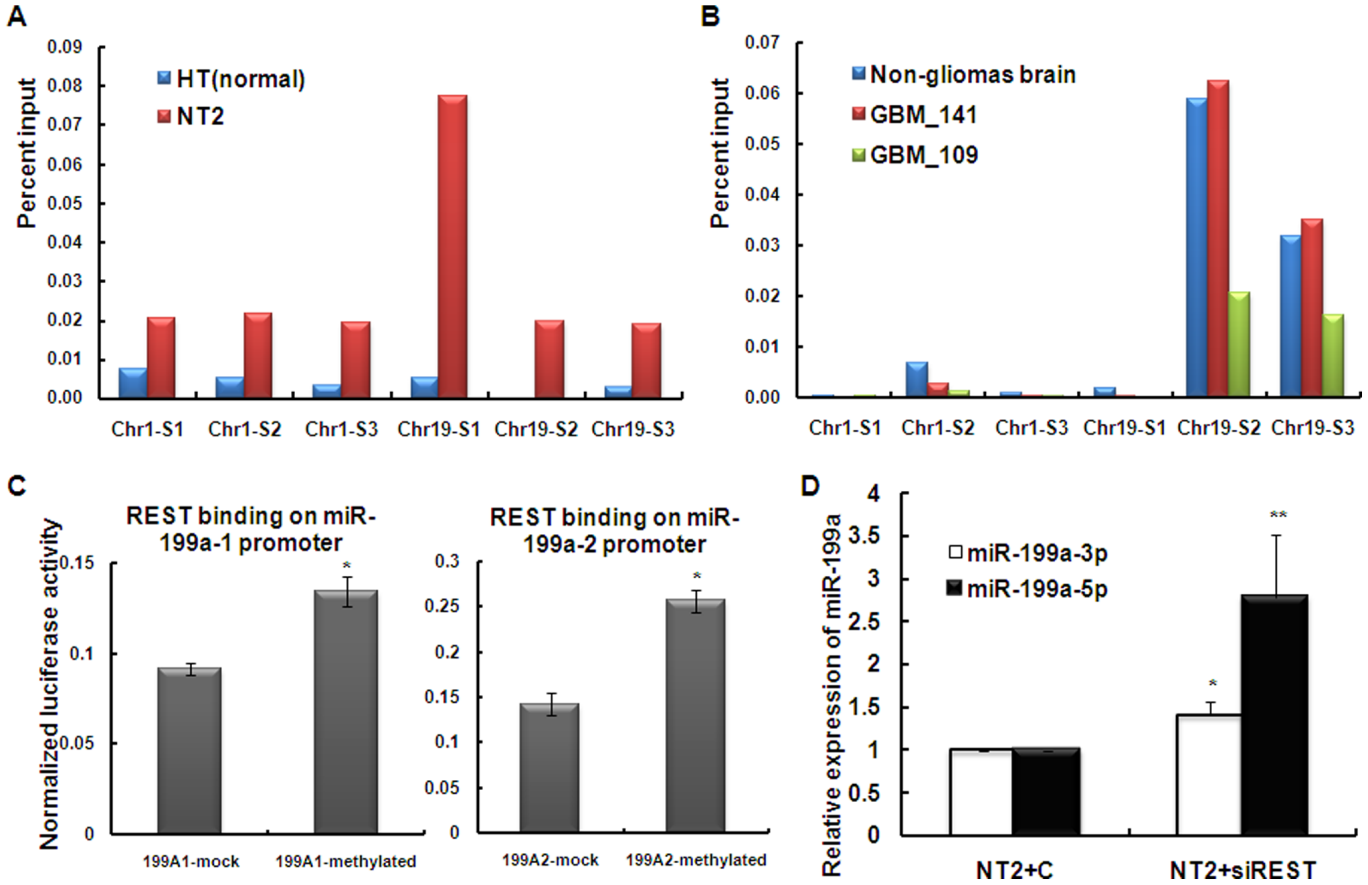
Function of REST on DNA methylation of miR-199a promoters. A). Chromatin immunoprecipitation (ChIP) confirmed the direct binding of transcription factor REST on both promoters of miR-199a in Chr1 and Chr19 in NT2 and HT cells. qPCR following ChIP showed that there were higher binding in NT2 cells compared to HT cells. S1, S2 and S3 are three consecutive segments of miR-199a promoters in Chr1 and Chr19 (Figure S1). There was binding of REST on Chr19-S2 in NT2 cells but not in HT cells. B). ChIP confirmed the direct binding of REST on one non-glioma brain tissue and two glioma tissues. C). *In vitro* methylation assay showed that REST preferentially bound to methylated miR-199a vectors than unmethylated vectors. Promoters of miR-199a-1 in Chr19 and miR-199a-2 in Chr1 were methylated *in vitro* and ligated into pGL3 luciferase vector (199A1-methylated and 199A2-methylated). Mock methylated (un-methylated) inserts were also ligated into pGL3 vector (199A1-mock and 199A2-mock). Ligated products were co-transfected with REST expressing vector into HEK-293T cells. Luciferase receptor assay was performed 48 hours after transfection. pGL4.73 vector was also transfected as normalization of the luciferase activity. *, p<0.05 luciferase activity compared methylated inserts with control. D). Reduced expression of REST resulted in increased expression of both miR-199a-3p and -5p in NT2 cells (Amount of siRNA of REST transfected: 30 nM). *, p<0.05 miR-199a-3p expression in NT2 cells with siREST transfection compared with control; **, p<0.05 miR-199a-5p expression in NT2 cells with siREST transfection compared with control.

In order to understand the regulation of DNA methylation of miR-199a promoters in both Chr1 and Chr19, we searched for possible regulator(s) for both loci. From UCSC genome browser (NCBI36/hg18), we discovered a common binding factor for both miR-199a-1 and miR-199a-2 promoters, named REST, from ENCODE transcription factor ChIP-seq results. We confirmed the binding of REST on the two promoters of miR-199a in both testis cells and brain tissues by ChIP (Figure 3A, 3B, Figure S2). qPCR followed ChIP showed that there were higher binding of REST in NT2 cells comparing to HT cells (Figure 3A, Figure S5), which may be related to the DNA methylation levels in these two cell types. While in brain tissues, REST was found to bind tightly to Chr19 in both non-gliomas brain and two gliomas tissues, but moderately bound to Chr1 in non-cancerous brain and almost no binding to Chr1 in gliomas (Figure 3B, Figure S5). The binding of REST to miR-199a promoters in brain tissues was also consistent with their DNA methylation status. In addition, *in vitro* methylation assay indicated a higher binding of REST to methylated promoters for both miR-199a-1 and -2 comparing to the un-methylation compartment (Figure 3C). The regulatory function of REST upon miR-199a expression was demonstrated by the up-regulation of both miR-199a-3p and -5p upon reduced expression of REST in NT2 cells (Figure 3D).

### Transcriptome changes upon miR-199a-5p in TGCTs

**Figure 4:**
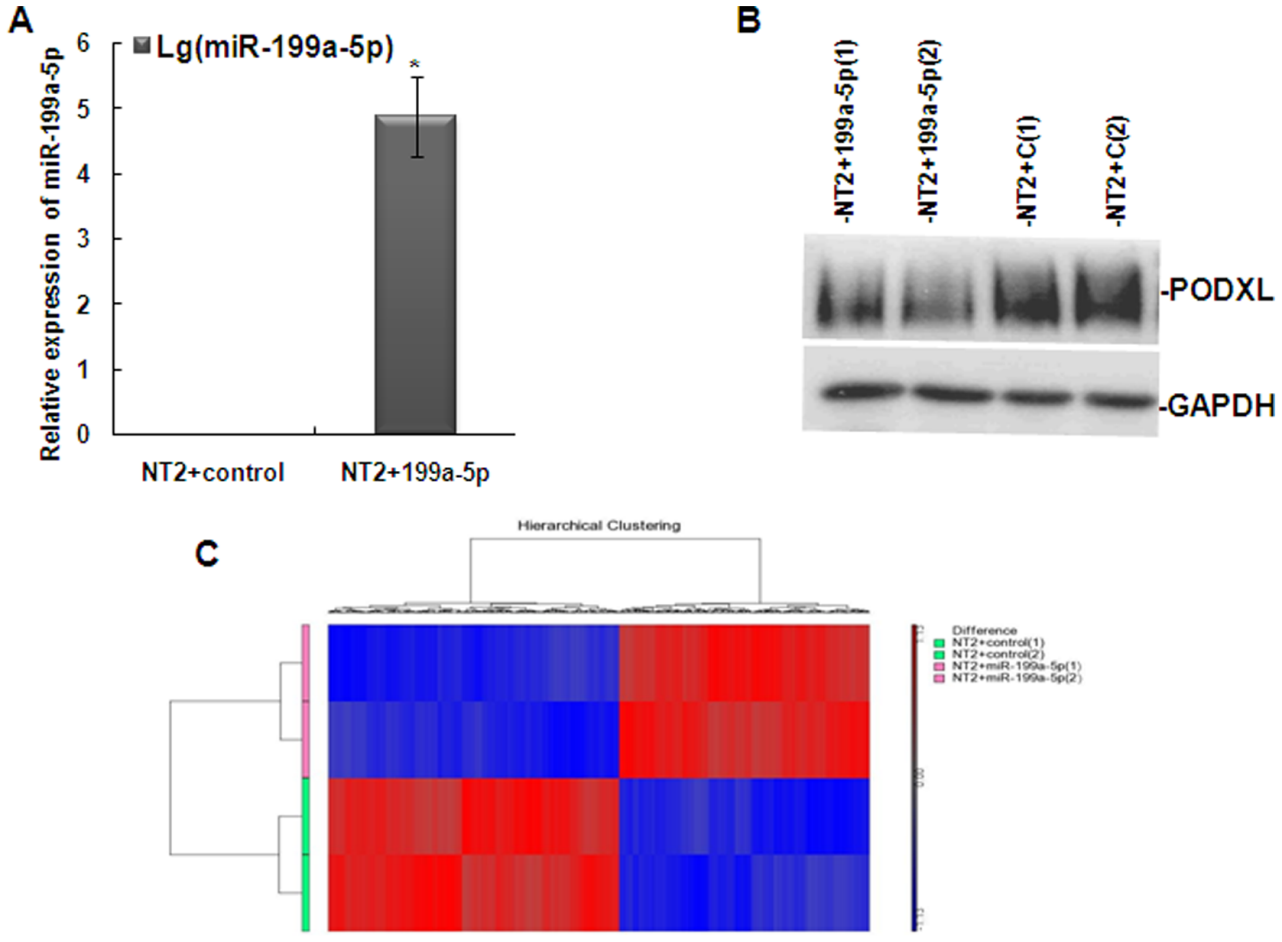
Identification of dysregulated genes after knock-in of miR-199a-5p in NT2 cells. A). qPCR showing more than four thousand fold changes of miR-199a-5p expression levels after transiently transfection of mimic of miR-199a-5p into NT2 cells comparing to NT2 cells transfected with scrambled control RNA. (Amount of miR-199a-5p mimics or scrambled control transfected: 25 nM). *, p<0.05 miR-199a-5p expression compared miR-199a-5p mimics transfection in NT2 cells with control. B). Western Blot showing reduced expression of PODXL, a confirmed direct target of miR-199a-5p in TGCTs after miR-199a-5p overexpression. C). Heatmap showing hierarchical clustering of differently expressed genes after miR-199a-5p transfection (Fold change>1.2, FDR<0.6).

Since the functions of a microRNA could only be exhibited through its targets, we tried to identify the direct target(s) of miR-199a-5p in order to understand its biological roles. Over-expression of miR-199a-5p was achieved by transfection of miR-199a-5p mimics into NT2 cells (Figure 4A). NT2 cells transfected with scrambled RNA fragment were used as controls. A previously identified target of miR-199a-5p in TGCTs, PODXL[23], showed reduced protein expression in miR-199a-5p transfected cells by Western blot (Figure 4B).

**Figure 5:**
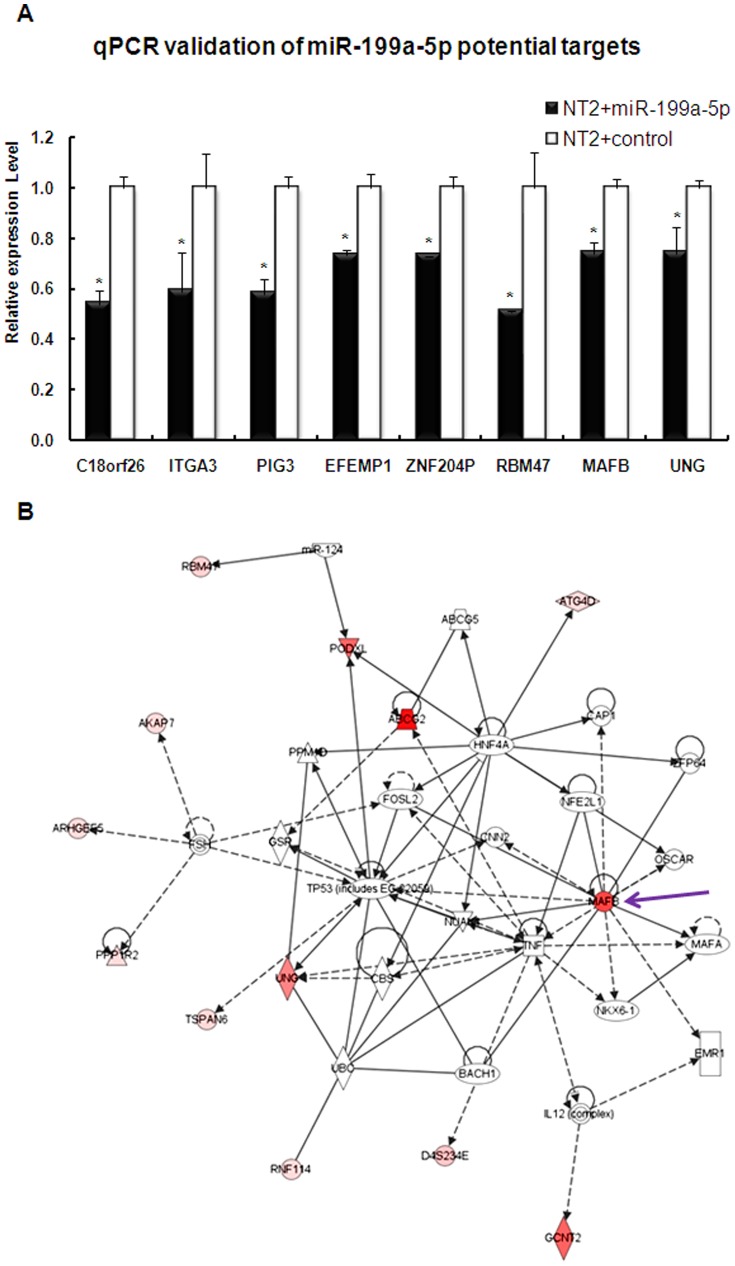
Validation of the expression arrays data by qPCR of selected genes. A). All these genes were potential direct targets of miR-199a-5p according to prediction programs, e.g. Targetscan and MiRanda. *, p<0.05 compared miR-199a-5p transfected group with the control. B) Ingenuity Pathway Analysis showed the relationship among the potential targets. “Master regulator” MAFB were selected for further studies (indicated by an arrow).

RNA from miR-199a-5p transfected cells and scrambled control transfected cells were isolated and purified for transcriptome studies by microarray expression chips (Figure 4C). The analysis was performed in duplication. A number of genes showed significant differential expression between the two groups (Table S2), with more genes showing down-regulation than up-regulation in miR-199a-5p transfected cells, consistent with the repression function of microRNA. Genes were selected for validation by qPCR according to the following criteria (Figure 5A): 1) Genes with significant reduced expression after miR-199a-5p transfection; 2) Genes predicted to be direct target of miR-199a-5p by prediction programs (e.g. Pictar, TargetScan, MiRanda). Selected genes were also analyzed by IPA to show their possible relationship among each other (Figure 5B).

### Identification of MAFB as a putative target of miR-199a-5p in TGCTs

One potential target, MAFB, was selected for further validation because: 1) Both expression array and qPCR revealed its reduced expression upon miR-199a-5p transfection; 2) Expression of MAFB was high in HT cells but much lower in NT2 cells (Figure S3); 3) The fact that MAFB occupies a central location in the pathway map generated suggests that it is likely to be a “master” in controlling other genes and act as a key regulator to exhibit functions of miR-199a-5p (Figure 5B).

**Figure 6:**
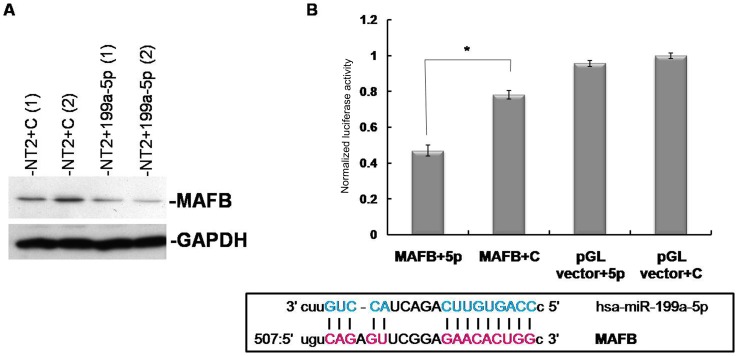
Validation of MAFB to be a direct target of miR-199a-5p in NT2 cells. A) Western blot showing expression decreased at protein level of MAFB after miR-199a-5p transfection; B) Luciferase receptor assay demonstrated direct binding between MAFB and miR-199a-5p. 3′-UTR of MAFB containing miR-199a-5p targeting site (MAFB) was cloned to the 3′-end of firefly luciferase (pGL vector). The plasmids were co-transfected with miR-199a-5p mimics (5p) or scrambled miRNA control. Lower panel showed the seed binding region between miR-199a-5p and 3′-UTR of MAFB (adapted from MiRanda database).

Western blotting showed that miR-199a-5p expression resulted in reduced MAFB protein level (Figure 6A). Direct interaction between miR-199a-5p and MAFB was demonstrated by reduced luciferase report activity when miR-199a-5p and the recombinant plasmid of 3′-UTR of MAFB linked to the 3′-end of firefly luciferase reporter (MAFB-pGL) were co-expressed in NT2 cells comparing to cells transfected with scrambled control (Figure 6B). These results indicated that MAFB could be a direct target of miR-199a-5p in TGCTs *in vitro*.

### MAFB is highly expressed in malignant testicular tumor and negatively correlated with miR-199a-5p

**Figure 7:**
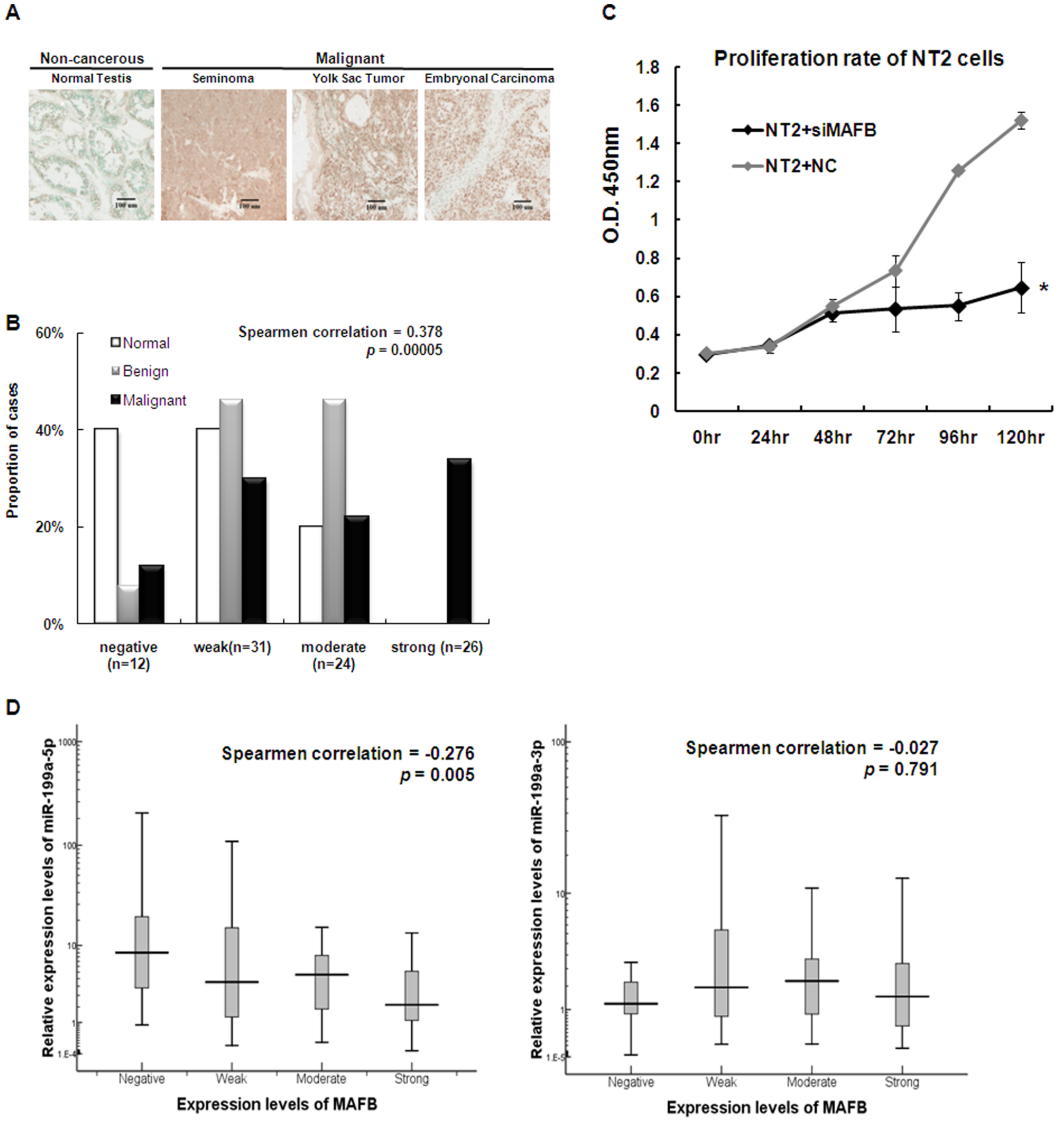
Functional studies of MAFB in TGCTs. A). Representative images of immunohistochemistry of MAFB in non-cancerous and malignant testicular tumor sections. B). Testis patient samples were divided into four groups(negative, weak, moderate or strong) based on the MAFB level and the proportion of different grades of tumors showing that MAFB protein is highly expressed in malignant testicular tumor. C) Depletion of MAFB by RNAi suppressed cell proliferation *in vitro* as revealed by CCK8 assay. *, p<0.05 siMAFB transfected NT2 cells compared with scrambled control transfected cells. D) Scatter plots of miR-199a-5p and miR-199a-3p expression against MAFB level. Expression of miR-199a-5p, but not -3p, correlates negatively with MAFB level (Negative: n = 12; Weak: n = 31; Moderate: n = 24; Strong: n = 26; miR-199a-5p: Spearman correlation = −0.276, *p* = 0.005; miR-199a-3p: Spearman correlation = −0.027, *p* = 0.791).

MAFB was further confirmed as a direct target of miR-199a-5p by studies with testis tissues. Immunostaining of MAFB on testis tissue arrays containing non-cancerous testis tissue and various types of testis tumors (including seminoma, yolk sac tumor and embryonal carcinoma) showed that MAFB expression was higher in cancerous tissues than in non-cancerous testis (Figure 7A). The low expression of MAFB in non-cancerous testis, the medium expression in benign tissue and the high expression in malignant testis were further confirmed by ranking correlation analysis (Spearman correlation factor = 0.378, p = 0.00005) (Figure 7B). In addition, in order to study the relationship between the expression of miR-199a-5p and MAFB, RNA isolated from the testis tissue arrays used for MAFB staining was subjected to Taqman qPCR. Expression of miR-199a-5p was statistically significantly reverse-correlated with expression of MAFB in testis tissues (Spearmen ranking correlation factor = −0.276, p = 0.005). However, correlation was not found between -3p and MAFB expression (Spearmen ranking correlation factor = −0.027, p = 0.791).

### MAFB knockdown suppresses tumor cell growth *in vitro*

Our previously studies showed anti-proliferation effect of miR-199a in TGCTs[23]. Here, we showed that the anti-proliferation function of miR-199a-5p may be realized through its target MAFB, as knock-down of MAFB expression in NT2 cells could significantly reduce the growth rate of cells (Figure 7C). The reduced proliferation maintained beyond 72 hours after transfection of MAFB siRNA.

## Discussion

miR-199a-2 first attracted our attention during the genome-wide methylation studies in TGCTs to uncover its hypermethylated status in tumor cells and tissues comparing to normal[10]. Similar observations were made by others in non-small cell lung cancer, colorectal cancer and breast cancer cell lines[27]. Previously we showed that expression of miR-199a was significantly lower in TGCTs comparing to normal testis cells. Upon de-methylation with 5-aza, expression of miR-199a was restored[10]. In addition, we showed the hypermethylated status of promoter of miR-199a-2 (91%) in Chr1 in TGCTs comparing to the hypomethylated promoter (0%) in HT cells by bisulfite sequencing. Here we showed that not only miR-199a-2 on Chr1, but also miR-199a-1 on Chr19 showed hypermethylation in NT2 cells. The two loci were regulated simultaneously in TGCTs.

To understand the biological roles of miR-199a in tumor, we compared the expression of miRNA-199a in TGCTs with that in malignant (grade IV) glioblastomas tissues in male patients. Contrary to the down-regulation of miR-199a in TGCTs, expression of miR-199a was up-regulated in gliomas (Figure 2C), and the elevated expression may be due to the hypomethylation of the promoter in Chr1 but not in Chr 19 (Fig 1C and 2B). Therefore, unlike the simultaneous regulation in TGCTs, the expressions of miR-199a in the two chromosomes were regulated differently in gliomas. Since miR-199a-2 resides within the DNM3OS gene and sometimes the expression of miR-199a-2 is regulated through the effects of transcription factor on DNM3OS [13], however, in the two cancer systems we studied here, we did not observe a correlation between DNM3OS and miR-199a expression (negative data not shown).

REST (RE-1 silencing transcription factor or NRSF, neuron-restrictive silencer factor) is a master repressor of neuronal genes[28]. One of its repression mechanisms involves its co-factors CoREST and the methyl DNA-binding protein MeCP2[29] that binds to methylated DNA. Here we demonstrated the directed binding of REST to both miR-199a-1 and -2 promoters in testis cells and in brain tissues by ChIP (Fig. 3A, 3B), and the degree of binding is related to the degree of methylation. In testis cells, REST bound much higher in miR-199-1 and -2 promoters in NT2 cells compared to normal testis fibroblasts. This was consistent with the much higher degree of DNA methylation in the former cell type comparing to the latter. Similarly in brain tissues, both non-gliomas brain and gliomas tissues showed hypermethylation of miR-199a-1 promoter, therefore a high REST binding. While for miR-199a-2 promoter, the ∼50% of DNA methylation of normal brain DNA corresponded to a moderate REST binding of Chr1, whereas the hypomethylation of gliomas tissues corresponded to a very low degree of REST binding.

In order to further establish the methylation status of miR-199a promoters versus the degree of REST binding and its potential effects on miR-199a expression, we performed *in vitro* methylation assay by simultaneous insertion of either methylated or un-methylated miR-199a promoter sequences in a luciferease reporter vector. Upon co-transfection of REST expression vector into HEK-293T cells, the higher luciferase activities of the methylated group compared to the un-methylated group indicated a higher binding of REST on methylated promoters for both miR-199a-1 and -2 (Figure 3C). In addition, we showed that reduced expression of REST could increase the expression of miR-199a (Figure 3D). These assays provided the evidence for a regulatory mechanism on miR-199a expression through REST binding to its promoters, and the possible relations of REST to DNA methylation.

Since the functions of a miRNA could only be realized through its targets, besides the upstream regulation of miR-199a, we aimed to understand its biological roles by studying its downstream targets. Previously in TGCTs, we showed that miR-199a behaved as a tumor-suppressor with anti-proliferation and anti-invasion effects[23]. Its anti-invasion effect was demonstrated to be realized through the direct target of miR-199a-5p, PODXL[23]. Here we identified another direct target of miR-199a-5p, MAFB (v-maf musculoaponeurotic fibrosarcoma oncogene homolog B (avian)), to explain its anti-proliferation effect.

In order to study the functions of miR-199a in TGCTs, we over-expressed miR-199a-5p in TGCT cells (NT2 cells) and examined the changes in transcriptome by microarray analysis. Most of the altered genes showed down-regulation (218 genes down-regulated, 91 genes up-regulated), consistent with the repression effect of miRNAs. Among the selected and q-PCR verified genes with reduced expression upon miR-199a-5p expression, we performed pathway analysis and identified MAFB as a “master” regulator (Figure 5B). MAFB is a basic leucine zipper (bZIP) transcription factor that controls hematopoietic myeloid differentiation. It could maintain the myelomonocytic phenotype by repressing ETS1-mediated transcription of erythroid-specific genes in myeloid cells[30], [31]. Together with other members from the MAF family, the carcinogenesis effects of MAFB may be implicated in 50% of human multiple myeloma[32].

Although well-studied in multiple myeloma, the function of MAFB in TGCTs has never been reported. Here we showed that upon knock-down by siRNA against MAFB, proliferation rate of NT2 cells decreased significantly compared to controls (Figure 7C). In addition, we demonstrated MAFB to be a direct target of miR-199a-5p with reduced expression of both mRNA and protein (Figure 5A, 6A) both in cells and in testis tissues (Figure 6A, 7A, 7B, 7D), and the direct binding between miR-199a-5p and the 3′-UTR of MAFB through luciferase receptor assay (Figure 6B). Therefore, the anti-proliferation effect of miR-199a may be realized through its repression of MAFB in TGCTs. Together with the anti-invasion effect mediated by the action of PODXL, miR-199a serves as a tumor-suppressor in TGCTs.

There is another miRNA, miR-199b, highly homologous to miRNA-199a[33]. miR-199b locates in Chromosome 9, being antisense of one intron of DNM1 while miR-199a-1 locates in Chromosome 19 and is within an intron of DNM2 and miR-199a-2 locates in Chromosome 1 and is within an intron of DNM3. Previous studies, mostly done in cancers, especially breast and bone cancers, suggested that only miR-199b-5p is functional[34]–[36]. miR-199b-5p has identical targets, e.g. HIF1a, PODXL, DDR1, etc, as miR-199a-5p, probably because their sequences are almost identical with only two different base pairs (miR-199a-5p: cccaguguucagacuaccuguuc; miR-199b-5p: cccaguguuuagacuaucuguuc)[37], [38]. In the case of the 3′UTR of MAFB, these two base pairs are not within the seed regions. Therefore, it is likely that miR-199b-5p also targets MAFB.

Using miR-199a as an example, the two genomic loci that give rise to mature miR-199a could be regulated simultaneously as in TGCTs, or could be regulated differently as in gliomas. The biological roles of miR-199a are further complicated by its various targets in different systems. We identified PODXL and MAFB as putative targets of miR-199a-5p in TGCTs and explained the anti-invasion and anti-proliferation effects of the miRNA mediated by these targets. However, in gliomas tissues, these two genes did not show expression correlations with miR-199a-5p (Figure S4), indicating that they may not be targets of miR-199a in gliomas. By studying the mechanisms that control the expressions of miR-199a and its various downstream targets, we hope to use miR-199a as a model to illustrate the complexity of miRNA biology.

## Materials and Methods

### Cell culture

NT2, HT and HEK-293T cell lines were purchased from ATCC (Manassas, VA, USA). NT2 and HT cells were cultured in Dulbecco'smodified Eagle's medium (Invitrogen, Carlsbad, CA, USA) supplemented with 10% fetal bovine serum. HEK-293T cells were cultured in Minimum Essential Medium(Invitrogen, Carlsbad, CA, USA) supplemented with 10% fetal bovine serum. All cells were maintained in a humidified incubator at 37°C with 5% CO_2_.

### Normal and tumor samples

Normal human brain DNA was purchased from BioChain (San Francisco, CA, USA). Human brain total RNA was purchased from Ambion (Austin, TX, USA). Glioblastomas patient samples and non-glioma brain tissues were collected in Prince of Wales Hospital, HKSAR and approved by Joint Chinese University of Hong Kong-New Territories East Cluster Clinical Research Ethics Committee. Written informed consent was obtained for research purposes. Testicular tumor tissue microarrays (TE2081) were purchased from US Biomax (Rockville, MD, USA). The panel includes 22 cases of normal, 13 cases of benign and 81cases of malignant tumors (50 seminomas and 31 nonseminomas). Patient information was summarized in Table S1.

### Bisulfite sequencing

Genomic DNA from cells or glioma samples was purified by PureLink Genome DNA kit (Invitrogen). For genome bisulphite sequencing, genomic DNA (500 ng) was treated with sodium bisulfite using EZ DNA Methylation-Gold Kit (Zymo Research, Irvine, CA, USA). Bisulfite converted DNA (50 ng) was used for PCR amplification using Platinum *Taq* DNA Polymerase (Invitrogen). The PCR product was TOPO-cloned (Invitrogen) and ∼10 positive clones were sequenced. Graphics of CpG methylation were generated by CpGviewer[39].

### RNA isolation, RT-PCR and quantitative real-time PCR (qPCR)

RNA from cultured cells and glioma tissues was isolated by Trizol Reagent (Invitrogen). RNA from formalin-fixed paraffin-embedded tissues was isolated by Recover All Total Nucleic Acid Isolation Kit (Ambion). For qPCR testing mRNA levels of genes, total RNA (500 ng) were reverse transcribed by PrimeScript RT Reagent Kit (TaKaRa, Dalian, China). All transcripts were quantified by quantitative PCR with Brilliant SYBR Green QPCR Master Mix (Applied Biosystems, Foster City, CA, USA) and a Light Cycler apparatus (Applied Biosystems, 7900HT), and the level of gene expression was normalized with GAPDH. Primer sequences are shown in Figure S3. For miRNA quantification, cDNA was synthesized from 10 ng of total RNA using miRNA-specific primers with TaqMan MicroRNA Reverse Transcription Kit (Applied Biosystems) and normalized by U6 (or hsa-miR-191 for results in Figure 7D). The level of expression of each target gene was then calculated as −2^ΔΔCt^. Primer sequences are listed in Table S3.

### Chromatin Immunoprecipitation (ChIP)

ChIP and qPCR were performed using MAGnify Chromatin Immunoprecipitation System (Invitrogen) according to the manufacturer's protocol. ChIP grade antibody REST was purchased from Merck Millipore. Generally, NT2 cells, HT cells or homogenized brain tissues were fixed by formaldehyde for a final concentration of 1%. Chromatin was sheared into 200–500 bp fragments using Bioruptor UCD-200 (Diagenode, Denville, NJ, US). 5 µL of sonicated cell or tissue lysates (concentration = 1 million cells per 50 µL lysis buffer), equivalent to 100 000 cells were used for each IP reaction. 10% of input amount (equivalent to 10 000 cells) was used as the input control for each sample. 2 µg of REST antibody or rabbit IgG (negative control) was used for each IP reaction for the binding of the chromatin lysates. After binding and repeated washing steps, the obtained DNA fragments were subjected to PCR using GoTaq DNA Polymerase (Promega) or qPCR with Brilliant SYBR Green QPCR Master Mix (Applied Biosystems) and a Light Cycler apparatus (Applied Biosystems, 7900HT). Sequences of primers used were illustrated in Figure S1. Calculation of relative binding of REST in each sample is as the following: qPCR reactions using 10-fold serial dilutions of the input control DNA were performed to determine the qPCR standard curve slope for each primer. Amplification efficiency (AE) was then calculated by the formula AE  =  10? (−1/slope). Percent input of each IP reaction was calculated by: 100×AÊ (Ct from input control – Ct from IP reaction).

### Luciferase receptor assay

The flanking sequence from 3′-UTR of MAFB containing predicted miRNA binding site was cloned to the pmirGLO Dual-Luciferase miRNA Target Expression Vector (Promega, Madison, WI, USA). A volume of 100 ng of pGL-MAFB vector or empty pGL vector was co-transfected with miR-199a-5p or scrambled control into NT2 cells (24-well format, six replicates for each combination). 48 h after transfection, cells were lysed and luciferase activity was measured by Luminometer (Promega) using the Dual-Luciferase Reporter Assay System Kit (Promega).

### Cloning of miR-199a promoter and *in vitro* methylation

The promoter region of miR-199a-1 (46:−670) and miR-199a-2 (+4:−370) were amplified by PCR and ligated to luciferase reporter vector pGL3-Basic (Promega). Primers sequences were illustrated in Figure S1 (“Full length” on Chr19 and Chr1, respectively). *In vitro* methylation was performed as previously described[40]. Briefly, promoters regions of miR-199a were restriction-cut from pGL3 construct, gel-purified and *in vitro* methylated by CpG methyltransferase M.SssI (New England Biolabs, Ipswich, MA, USA). Methylation of promoter was verified by methylation sensitive restriction enzyme BstUI. Either methylated or unmethylated promoter DNA was ligated to the luciferase vector in a molar ratio of 1∶1. The ligated products (3 mg) were used directly for transfection together with REST expression vector pEZ-REST_ORF to HEK-293T cells.

### Antibody for Western blot

Primary antibodies used were as follows: PODXL (clone 3D3, Santa Cruz Biotechnology, Santa Cruz, CA, USA, 1∶200 dilution); MAFB (Atlas Antibodies, Stockholm, Sweden, 1∶200 dilution); GAPDH (Abcam, 1∶10000 dilution). Secondary antibodies (1∶10000) were purchased from Bio-Rad (Hercules, CA, USA).

### Cell transfection

miR-199a-5p mimics and miRNA scrambled control were purchased from Ambion. siRNA of MAFB was designed by GenePharma Company (Shanghai, China). Vectors (pmirGLO and pGL3 from Promega, pEZ-REST_ORF from GeneCopoeia) or miRNA molecules were transfected with indicated amount into cells using Lipofectamine2000 (Invitrogen). Cells were harvest for RNA or protein extraction 48 h after transfection.

### Expression microarray and IPA analysis

Total RNA from transfected NT2 cells were isolated by Trizol reagent (Invitrogen), DNaseI-treated and purified by PureLink RNA mini kit (Ambion) and analyzed using Bioanalyzer (Agilent, Santa Clara, CA, USA). RNA was amplified by Ambion WT Expression Kit. The resulting cRNA was labeled by GeneChip WT Terminal Labeling and Control Kit (Affymetrix, Santa Clara, CA, USA) and hybridized to GeneChip Human Gene 1.0 ST array (Affymetrix). The raw data was normalized by robust multiarray average algorithm and analyzed in Partek Genomics Suite Software. Differential gene expression was evaluated using one-way ANOVA and differentially expressed genes were confirmed by real-time PCR (Table S2). Select group of genes with significantly repressed expression after miR-199a-5p transfection were subjected for pathway analysis using IPA program. Data from expression array will be uploaded to GEO after acceptance of the manuscript.

### Immunohistochemistry (IHC)

Formalin-fixed paraffin-embeddedtissue arrays (Biomax) were de-parafinized in xylenes and hydrated in a gradual series of ethanol. Antigen retrieval was done by heating the slides in citrate buffer at 100°C for 10 minutes. The slides were probed with anti-MAFB antibody (Atlas Antibodies, Stockholm, Sweden, 1∶400 dilution) overnight at 4°C. Signal was developed using DAB Histochemistry Kit (Invitrogen). Cells were counter stained with 0.5% methyl green in acetate buffer. Expression of MAFB was scored as 0 (negative: no observable positive), 1 (weak: >0-25% positive), 2 (moderate: >25–75% positive) and 3 (strong: >75% positive) by two experienced scientists separately. Triplicate experiments were performed.

### Proliferation rate assay

Cell viability and proliferation were measured with a Cell Counting KIT-8 (CCK-8, Dojindo, Shanghai, China). After 48 h after transfection of NT2 cells with siRNA against MAFB or scrambled control, cells were incubated in CCK-8 solution in a 5% CO_2_ incubator at 37°C for 2 h. The intense orange-colored formazan derivative formed by cell metabolism is soluble in the culture medium. The absorbance was measured at 450 nm with a reference wavelength of 650 nm. Cell number was correlated to optical density (OD).

### Statistical analysis

Difference of qPCR expression, cell proliferation rate and luciferase receptor activity was analyzed by two-tailed Student's t-test. The correlations between MAFB expression and miR-199a-3p or -5p expression, MAFB expression and tumor malignancy were analyzed by Spearman's rank correlation. P<0.05 is considered statistically significant.

## Supporting Information

Figure S1 **Primer sequences for the qPCR followed ChIP assays.** For both promoters of miR-199a-1 and miR-199a-2, sequences in underlined bold black are pairs of primers for full length amplification. Both full lengths were divided into three consecutive segments. Pairs of primers for segment 1 (S1) are highlighted in red, segment 2 (S2) in green and segment 3 (S3) in blue.
(JPG)

Figure S2 **PCR followed by ChIP assays.** For each sample, 24 PCRs were performed using 8 pairs of primers (sequences shown in Figure S1): A1-A3, primer pair miR-199a-1-full-length; B1-B3, primer pair miR-199a-1-S1; C1-C3, primer pair miR-199a-1-S2; D1-D3, primer pair miR-199a-1-S3; E1-E3, primer pair miR-199a-2-S1; F1-F3, primer pair miR-199a-2-S2; G1-G3, primer pair miR-199a-2-S3; H1-H3, primer pair miR-199a-2-full-length. Wells labeled with 1 used templates from ChIP with REST antibody, wells labeled with 2 used templates from ChIP with rabbit IgG antibody as negative control, and wells labeled with 3 used templates from imput control of each sample.
(JPG)

Figure S3 **Detection of expression levels of MAFB in NT2 cells and HT cells by qPCR.**
(JPG)

Figure S4 **Detection of expression levels of MAFB and PODXL by qPCR in gliomas tissues and normal brain.**
(JPG)

Figure S5 **Relative expression of REST in brain tissues (A) and testis cells (B).**
(JPG)

Table S1 **Summary of clinical samples.**
(DOCX)

Table S2 **Dysregulated genes list after miR-199a-5p over-expression in NT2 cells.**
(XLSX)

Table S3 **Primer sequences.**
(XLSX)
