# Supplementary figures and images for: Molecular Mechanisms of Regulation and Action of microRNA-199a in Testicular Germ Cell Tumor and Glioblastomas
Source: PLoS One. 2013 Dec 31;8(12):e83980. doi: 10.1371/journal.pone.0083980 (PMC3877122; doi:10.1371/journal.pone.0083980)

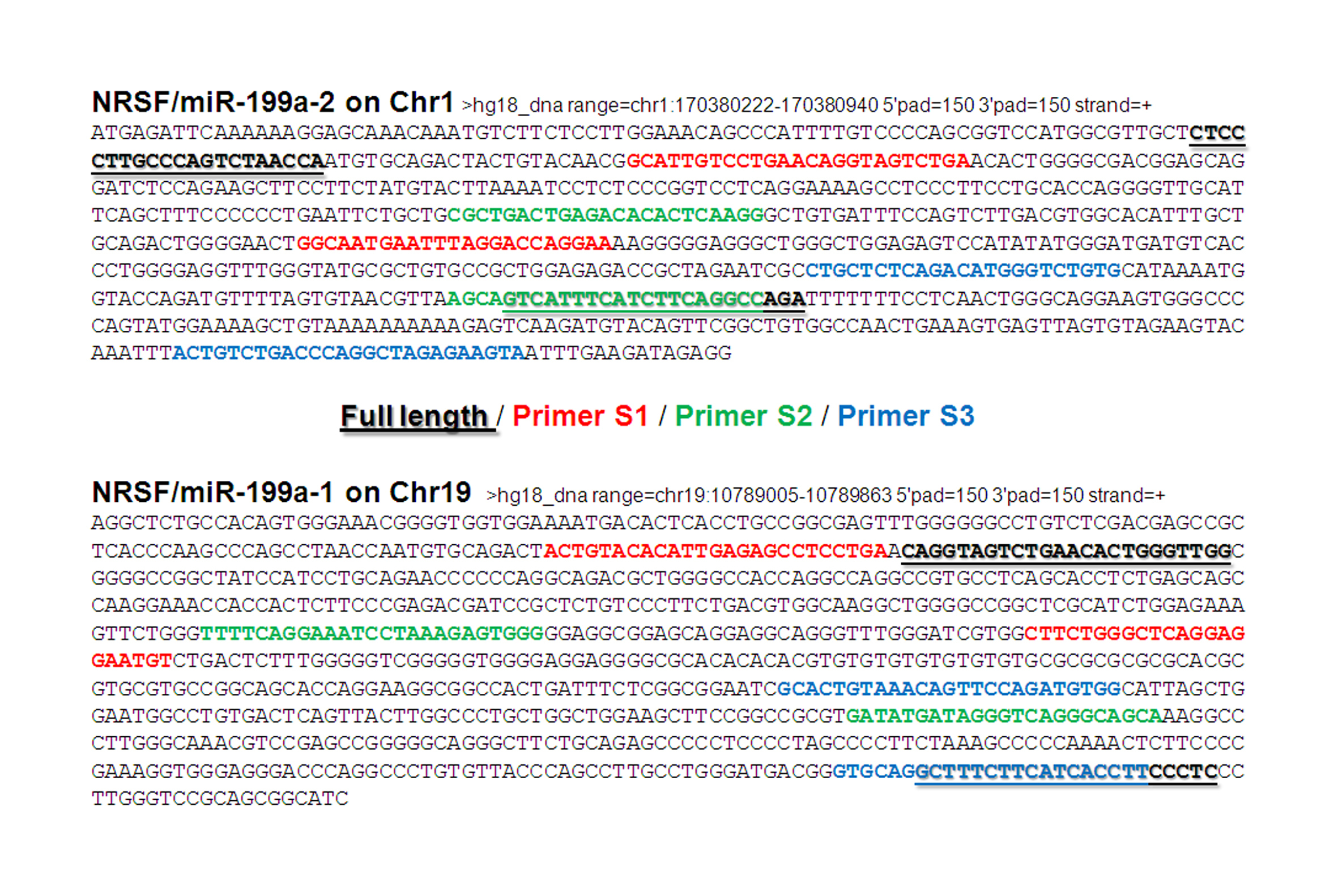

Supplement: Figure S1 — Primer sequences for the qPCR followed ChIP assays. For both promoters of miR-199a-1 and miR-199a-2, sequences in underlined bold black are pairs of primers for full length amplification. Both full lengths were divided into three consecutive segments. Pairs of primers for segment 1 (S1) are highlighted in red, segment 2 (S2) in green and segment 3 (S3) in blue. (JPG) [file pone.0083980.s001.jpg]

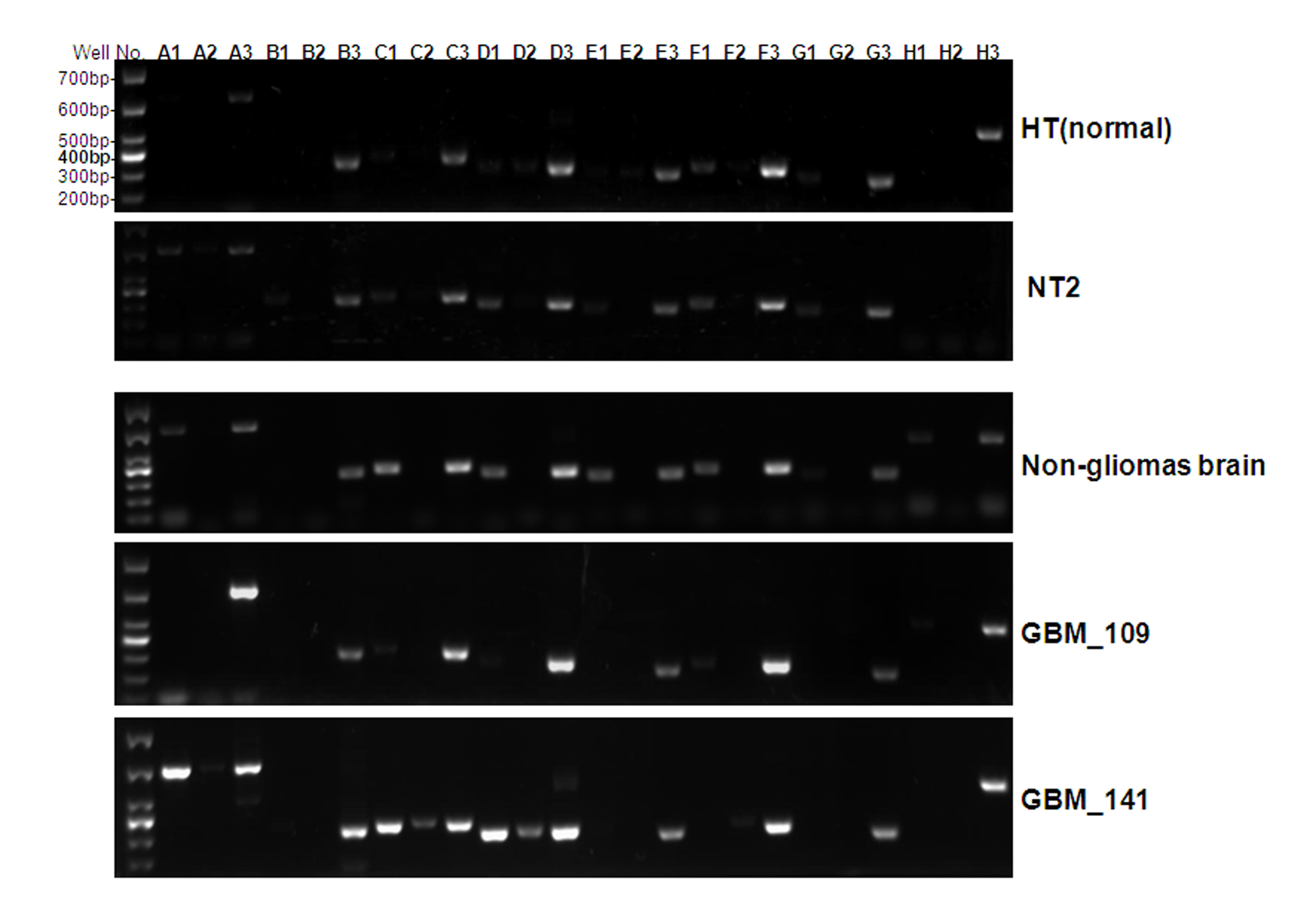

Supplement: Figure S2 — PCR followed by ChIP assays. For each sample, 24 PCRs were performed using 8 pairs of primers (sequences shown in Figure S1): A1-A3, primer pair miR-199a-1-full-length; B1-B3, primer pair miR-199a-1-S1; C1-C3, primer pair miR-199a-1-S2; D1-D3, primer pair miR-199a-1-S3; E1-E3, primer pair miR-199a-2-S1; F1-F3, primer pair miR-199a-2-S2; G1-G3, primer pair miR-199a-2-S3; H1-H3, primer pair miR-199a-2-full-length. Wells labeled with 1 used templates from ChIP with REST antibody, wells labeled with 2 used templates from ChIP with rabbit IgG antibody as negative control, and wells labeled with 3 used templates from imput control of each sample. (JPG) [file pone.0083980.s002.jpg]

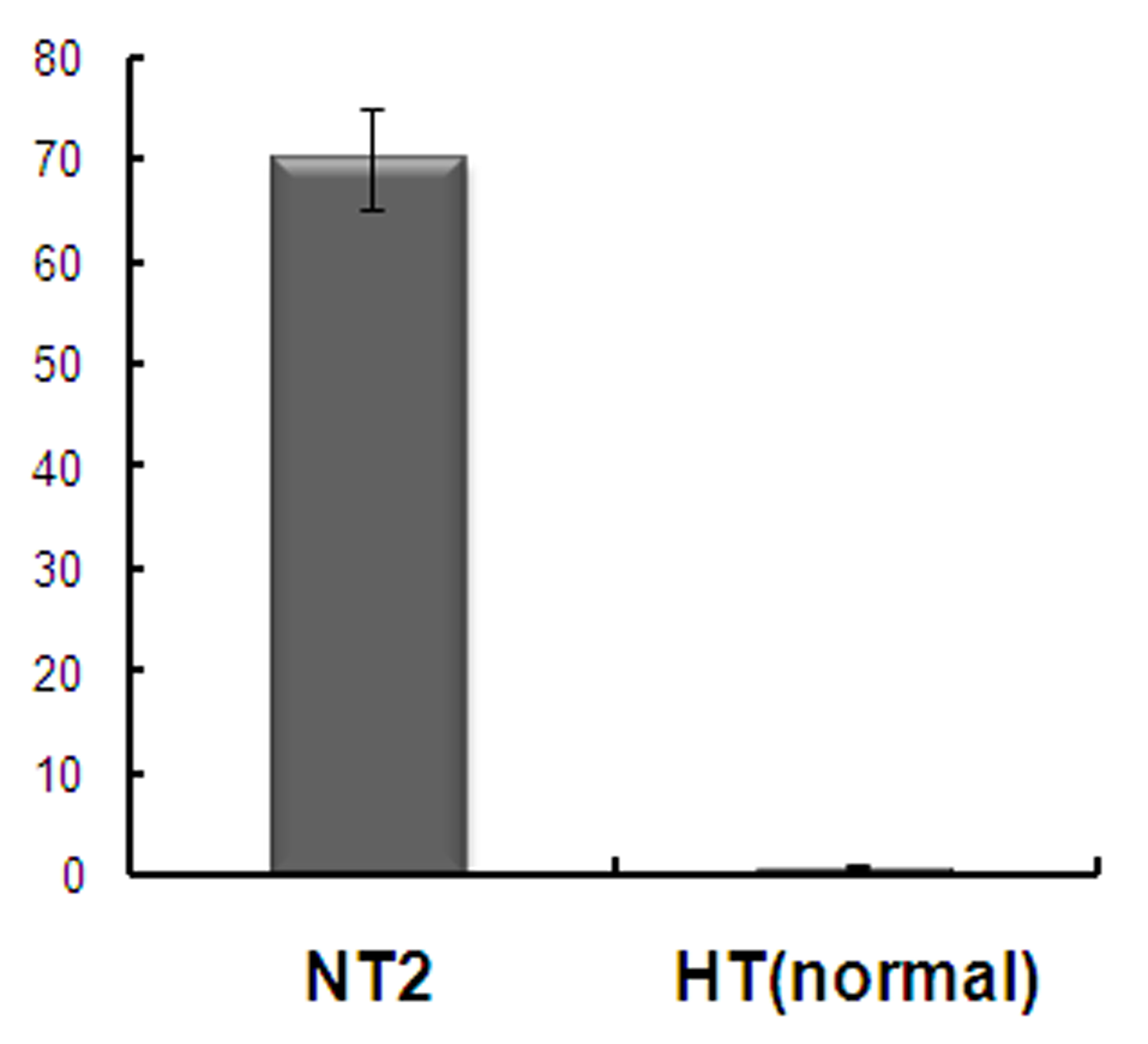

Supplement: Figure S3 — Detection of expression levels of MAFB in NT2 cells and HT cells by qPCR. (JPG) [file pone.0083980.s003.jpg]

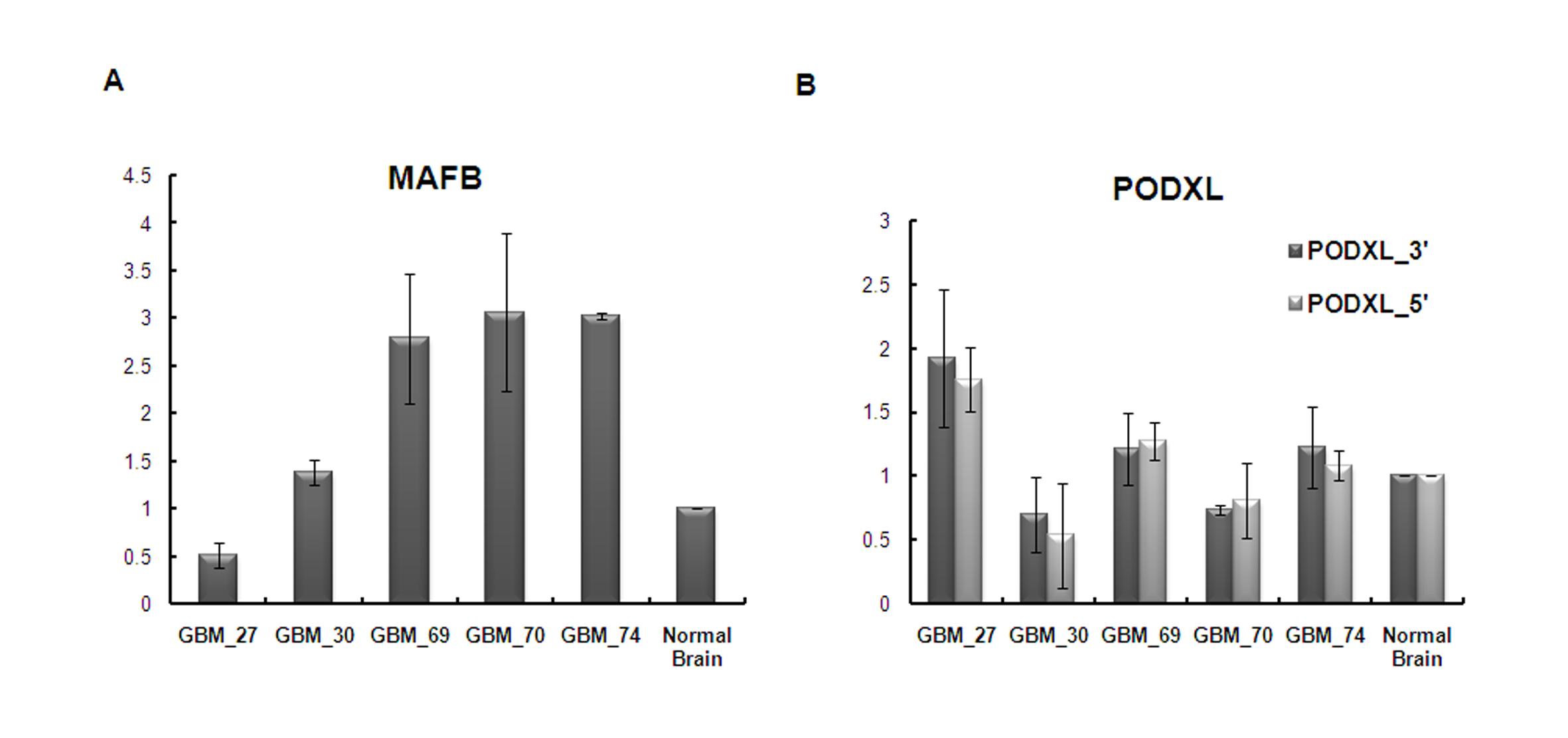

Supplement: Figure S4 — Detection of expression levels of MAFB and PODXL by qPCR in gliomas tissues and normal brain. (JPG) [file pone.0083980.s004.jpg]

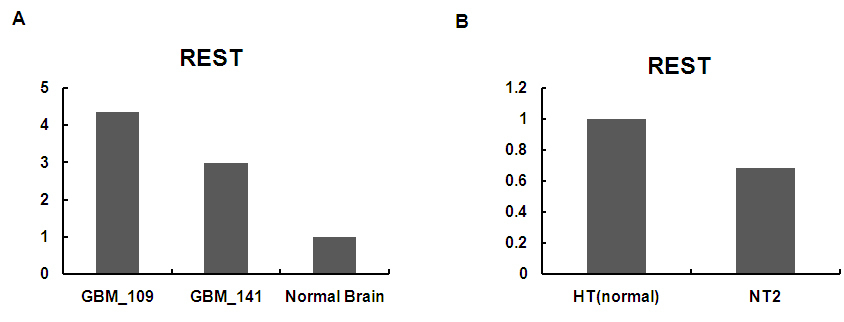

Supplement: Figure S5 — Relative expression of REST in brain tissues (A) and testis cells (B). (JPG) [file pone.0083980.s005.jpg]
