# Supplementary material for: Molecular Mechanisms of Regulation and Action of microRNA-199a in Testicular Germ Cell Tumor and Glioblastomas
Source: PLoS One. 2013 Dec 31;8(12):e83980. doi: 10.1371/journal.pone.0083980 (PMC3877122; doi:10.1371/journal.pone.0083980)
Supplement: Table S1 — Summary of clinical samples. (DOCX) [file pone.0083980.s006.docx]

**Supplementary Table 1** Summary of clinical samples

**Case summary for patients illustrated in Figure 2 and Figure 3**

| Case# | GENDER | AGE | GLIOMA STAGE | COMMENTS |
| --- | --- | --- | --- | --- |
| 27 | M | 63 | IV | Left temporal lobe, left parietal lobe glioblastoma mutiforme |
| 30 | M | 57 | IV | Right frontal lobe glioblastoma |
| 69 | M | 65 | IV | Left occipital lobe glioblastoma |
| 70 | M | 67 | IV | Left temporoparietal lesion, craniotomy |
| 74 | M | 66 | IV | Left frontal glioblastoma |
| 109 | M | 64 | IV | Left temporal lobe, left parietal lobe glioblastoma mutiforme |
| 141 | M | 65 | IV | Left occipital lobe glioblastoma |
| Normal Brain 340 | M | 33 | II-III | Normal tissue from a patient with left insular oligodendroglioma |
| Non-gliomas | F | 30 | N/A | Right frontal metastatic adenocarcinoma consistent with lung primary |

**Case summary of testis tissues in Figure 7**

| Array# | Position | Sex | Age | Organ | Pathology | TNM | Type |
| --- | --- | --- | --- | --- | --- | --- | --- |
| TE2081 | A1 | M | 59 | Testis | Seminoma | T4N0M0 | Malignant |
| TE2081 | A2 | M | 59 | Testis | Seminoma | T4N0M0 | Malignant |
| TE2081 | A3 | M | 52 | Testis | Seminoma | T2N0M0 | Malignant |
| TE2081 | A4 | M | 52 | Testis | Seminoma | T2N0M0 | Malignant |
| TE2081 | A5 | M | 31 | Testis | Seminoma | T1N0M0 | Malignant |
| TE2081 | A6 | M | 31 | Testis | Seminoma | T1N0M0 | Malignant |
| TE2081 | A7 | M | 38 | Testis | Seminoma | T1N0M0 | Malignant |
| TE2081 | A8 | M | 38 | Testis | Seminoma | T1N0M0 | Malignant |
| TE2081 | A9 | M | 45 | Testis | Seminoma | T1N0M0 | Malignant |
| TE2081 | A10 | M | 45 | Testis | Seminoma | T1N0M0 | Malignant |
| TE2081 | A11 | M | 52 | Testis | Seminoma | T2N0M0 | Malignant |
| TE2081 | A12 | M | 52 | Testis | Seminoma | T2N0M0 | Malignant |
| TE2081 | A13 | M | 32 | Testis | Seminoma | T1N0M0 | Malignant |
| TE2081 | A14 | M | 32 | Testis | Seminoma | T1N0M0 | Malignant |
| TE2081 | A15 | M | 40 | Testis | Seminoma | T1N0M0 | Malignant |
| TE2081 | A16 | M | 40 | Testis | Seminoma | T1N0M0 | Malignant |
| TE2081 | B1 | M | 36 | Testis | Seminoma | T2N0M0 | Malignant |
| TE2081 | B2 | M | 36 | Testis | Seminoma | T2N0M0 | Malignant |
| TE2081 | B3 | M | 70 | Testis | Seminoma | T1N0M0 | Malignant |
| TE2081 | B4 | M | 70 | Testis | Seminoma (fibrous tissue and blood vessel) | T1N0M0 | Malignant |
| TE2081 | B5 | M | 61 | Testis | Seminoma | T1N0M0 | Malignant |
| TE2081 | B6 | M | 61 | Testis | Seminoma | T1N0M0 | Malignant |
| TE2081 | B7 | M | 38 | Testis | Seminoma | T1N0M0 | Malignant |
| TE2081 | B8 | M | 38 | Testis | Seminoma | T1N0M0 | Malignant |
| TE2081 | B9 | M | 30 | Testis | Seminoma | T1N0M0 | Malignant |
| TE2081 | B10 | M | 30 | Testis | Seminoma | T1N0M0 | Malignant |
| TE2081 | B11 | M | 42 | Testis | Seminoma | T2N0M0 | Malignant |
| TE2081 | B12 | M | 42 | Testis | Seminoma | T2N0M0 | Malignant |
| TE2081 | B13 | M | 44 | Testis | Seminoma | T1N0M0 | Malignant |
| TE2081 | B14 | M | 44 | Testis | Seminoma | T1N0M0 | Malignant |
| TE2081 | B15 | M | 35 | Testis | Seminoma | T4N0M0 | Malignant |
| TE2081 | B16 | M | 35 | Testis | Seminoma | T4N0M0 | Malignant |
| TE2081 | C1 | M | 34 | Testis | Seminoma | T1N0M0 | Malignant |
| TE2081 | C2 | M | 34 | Testis | Seminoma | T1N0M0 | Malignant |
| TE2081 | C3 | M | 38 | Testis | Seminoma | T2N0M0 | Malignant |
| TE2081 | C4 | M | 38 | Testis | Seminoma | T2N0M0 | Malignant |
| TE2081 | C5 | M | 33 | Testis | Seminoma (sparse) | T1N0M0 | Malignant |
| TE2081 | C6 | M | 33 | Testis | Seminoma | T1N0M0 | Malignant |
| TE2081 | C7 | M | 39 | Testis | Seminoma | T2N0M0 | Malignant |
| TE2081 | C8 | M | 39 | Testis | Seminoma | T2N0M0 | Malignant |
| TE2081 | C9 | M | 33 | Testis | Seminoma | T1N0M0 | Malignant |
| TE2081 | C10 | M | 33 | Testis | Seminoma | T1N0M0 | Malignant |
| TE2081 | C11 | M | 44 | Testis | Seminoma | T2N0M0 | Malignant |
| TE2081 | C12 | M | 44 | Testis | Seminoma | T2N0M0 | Malignant |
| TE2081 | C13 | M | 36 | Testis | Seminoma | T1N0M0 | Malignant |
| TE2081 | C14 | M | 36 | Testis | Seminoma | T1N0M0 | Malignant |
| TE2081 | C15 | M | 30 | Testis | Seminoma | T2N0M0 | Malignant |
| TE2081 | C16 | M | 30 | Testis | Seminoma with necrosis | T2N0M0 | Malignant |
| TE2081 | D1 | M | 29 | Testis | Seminoma | T2N0M0 | Malignant |
| TE2081 | D2 | M | 29 | Testis | Seminoma | T2N0M0 | Malignant |
| TE2081 | D3 | M | 44 | Testis | Seminoma | T1N0M0 | Malignant |
| TE2081 | D4 | M | 44 | Testis | Seminoma | T1N0M0 | Malignant |
| TE2081 | D5 | M | 30 | Testis | Seminoma | T2N0M0 | Malignant |
| TE2081 | D6 | M | 30 | Testis | Seminoma | T2N0M0 | Malignant |
| TE2081 | D7 | M | 50 | Testis | Seminoma | T2N0M1 | Malignant |
| TE2081 | D8 | M | 50 | Testis | Seminoma (necrosis) | T2N0M1 | Malignant |
| TE2081 | D9 | M | 40 | Testis | Seminoma (chronic inflammation of fibrous tissue) | T1N0M0 | Malignant |
| TE2081 | D10 | M | 40 | Testis | Seminoma (chronic inflammation of fibrous tissue) | T1N0M0 | Malignant |
| TE2081 | D11 | M | 43 | Testis | Seminoma | T1N0M0 | Malignant |
| TE2081 | D12 | M | 43 | Testis | Seminoma | T1N0M0 | Malignant |
| TE2081 | D13 | M | 48 | Testis | Seminoma | T1N0M0 | Malignant |
| TE2081 | D14 | M | 48 | Testis | Seminoma | T1N0M0 | Malignant |
| TE2081 | D15 | M | 40 | Testis | Seminoma | T2N0M0 | Malignant |
| TE2081 | D16 | M | 40 | Testis | Seminoma | T2N0M0 | Malignant |
| TE2081 | E1 | M | 55 | Testis | Seminoma | T4N0M0 | Malignant |
| TE2081 | E2 | M | 55 | Testis | Seminoma | T4N0M0 | Malignant |
| TE2081 | E3 | M | 61 | Testis | Seminoma with necrosis | T4N0M0 | Malignant |
| TE2081 | E4 | M | 61 | Testis | Seminoma | T4N0M0 | Malignant |
| TE2081 | E5 | M | 28 | Testis | Seminoma | T2N0M0 | Malignant |
| TE2081 | E6 | M | 28 | Testis | Seminoma | T2N0M0 | Malignant |
| TE2081 | E7 | M | 56 | Testis | Seminoma | T2N0M0 | Malignant |
| TE2081 | E8 | M | 56 | Testis | Seminoma | T2N0M0 | Malignant |
| TE2081 | E9 | M | 34 | Testis | Seminoma | T1N0M0 | Malignant |
| TE2081 | E10 | M | 34 | Testis | Seminoma | T1N0M0 | Malignant |
| TE2081 | E11 | M | 28 | Testis | Seminoma | T4N0M0 | Malignant |
| TE2081 | E12 | M | 28 | Testis | Seminoma (chronic inflammation of testicular tissue sparse) | T4N0M0 | Malignant |
| TE2081 | E13 | M | 52 | Testis | Seminoma | T2N0M0 | Malignant |
| TE2081 | E14 | M | 52 | Testis | Seminoma | T2N0M0 | Malignant |
| TE2081 | E15 | M | 47 | Testis | Seminoma | T1N0M0 | Malignant |
| TE2081 | E16 | M | 47 | Testis | Seminoma | T1N0M0 | Malignant |
| TE2081 | F1 | M | 41 | Testis | Seminoma | T1N0M0 | Malignant |
| TE2081 | F2 | M | 41 | Testis | Seminoma | T1N0M0 | Malignant |
| TE2081 | F3 | M | 15 | Testis | Seminoma | T1N0M0 | Malignant |
| TE2081 | F4 | M | 15 | Testis | Seminoma | T1N0M0 | Malignant |
| TE2081 | F5 | M | 38 | Testis | Seminoma | T2N0M0 | Malignant |
| TE2081 | F6 | M | 38 | Testis | Seminoma | T2N0M0 | Malignant |
| TE2081 | F7 | M | 50 | Testis | Seminoma | T1N0M0 | Malignant |
| TE2081 | F8 | M | 50 | Testis | Seminoma | T1N0M0 | Malignant |
| TE2081 | F9 | M | 39 | Testis | Seminoma | T1N0M0 | Malignant |
| TE2081 | F10 | M | 39 | Testis | Seminoma | T1N0M0 | Malignant |
| TE2081 | F11 | M | 33 | Testis | Seminoma | T1N0M0 | Malignant |
| TE2081 | F12 | M | 33 | Testis | Seminoma | T1N0M0 | Malignant |
| TE2081 | F13 | M | 2 | Testis | Yolk sac tumor | T1N0M0 | Malignant |
| TE2081 | F14 | M | 2 | Testis | Yolk sac tumor | T1N0M0 | Malignant |
| TE2081 | F15 | M | 52 | Testis | Yolk sac tumor | T1N0M0 | Malignant |
| TE2081 | F16 | M | 52 | Testis | Yolk sac tumor | T1N0M0 | Malignant |
| TE2081 | G1 | M | 8 Month. | Testis | Yolk sac tumor | T2N0M0 | Malignant |
| TE2081 | G2 | M | 8 Month. | Testis | Yolk sac tumor | T2N0M0 | Malignant |
| TE2081 | G3 | M | 2 | Testis | Yolk sac tumor | T1N0M0 | Malignant |
| TE2081 | G4 | M | 2 | Testis | Yolk sac tumor | T1N0M0 | Malignant |
| TE2081 | G5 | M | 30 | Testis | Yolk sac tumor | T1N0M0 | Malignant |
| TE2081 | G6 | M | 30 | Testis | Yolk sac tumor | T1N0M0 | Malignant |
| TE2081 | G7 | M | 32 | Testis | Yolk sac tumor | T2N0M0 | Malignant |
| TE2081 | G8 | M | 32 | Testis | Yolk sac tumor | T2N0M0 | Malignant |
| TE2081 | G9 | M | 23 | Testis | Yolk sac tumor | T1N0M0 | Malignant |
| TE2081 | G10 | M | 23 | Testis | Yolk sac tumor | T1N0M0 | Malignant |
| TE2081 | G11 | M | 2 | Testis | Yolk sac tumor | T1N0M0 | Malignant |
| TE2081 | G12 | M | 2 | Testis | Yolk sac tumor | T1N0M0 | Malignant |
| TE2081 | G13 | M | 25 | Testis | Embryonal carcinoma | T4N1M0 | Malignant |
| TE2081 | G14 | M | 25 | Testis | Embryonal carcinoma | T4N1M0 | Malignant |
| TE2081 | G15 | M | 16 | Testis | Embryonal carcinoma | T1N0M0 | Malignant |
| TE2081 | G16 | M | 16 | Testis | Embryonal carcinoma | T1N0M0 | Malignant |
| TE2081 | H1 | M | 30 | Testis | Embryonal carcinoma | T1N0M0 | Malignant |
| TE2081 | H2 | M | 30 | Testis | Embryonal carcinoma | T1N0M0 | Malignant |
| TE2081 | H3 | M | 43 | Testis | Embryonal carcinoma | T2N0M0 | Malignant |
| TE2081 | H4 | M | 43 | Testis | Embryonal carcinoma | T2N0M0 | Malignant |
| TE2081 | H5 | M | 36 | Testis | Embryonal carcinoma | T1N0M0 | Malignant |
| TE2081 | H6 | M | 36 | Testis | Embryonal carcinoma | T1N0M0 | Malignant |
| TE2081 | H7 | M | 30 | Testis | Embryonal carcinoma | T2N0M0 | Malignant |
| TE2081 | H8 | M | 30 | Testis | Embryonal carcinoma | T2N0M0 | Malignant |
| TE2081 | H9 | M | 32 | Testis | Embryonal carcinoma (sparse) | T2N0M0 | Malignant |
| TE2081 | H10 | M | 32 | Testis | Embryonal carcinoma (testicular tissue sparse with atrophy) | T2N0M0 | Malignant |
| TE2081 | H11 | M | 24 | Testis | Embryonal carcinoma | T1N0M0 | Malignant |
| TE2081 | H12 | M | 24 | Testis | Embryonal carcinoma (fibrous tissue with necrosis) | T1N0M0 | Malignant |
| TE2081 | H13 | M | 21 | Testis | Embryonal carcinoma | T2N0M0 | Malignant |
| TE2081 | H14 | M | 21 | Testis | Embryonal carcinoma | T2N0M0 | Malignant |
| TE2081 | H15 | M | 26 | Testis | Embryonal carcinoma (sparse) with necrosis | T1N0M0 | Malignant |
| TE2081 | H16 | M | 26 | Testis | Embryonal carcinoma | T1N0M0 | Malignant |
| TE2081 | I1 | M | 30 | Testis | Embryonal carcinoma (atrophy) | T1N0M0 | Malignant |
| TE2081 | I2 | M | 30 | Testis | Embryonal carcinoma | T1N0M0 | Malignant |
| TE2081 | I3 | M | 18 | Testis | Embryonal carcinoma | T3N0M0 | Malignant |
| TE2081 | I4 | M | 18 | Testis | Embryonal carcinoma | T3N0M0 | Malignant |
| TE2081 | I5 | M | 38 | Testis | Embryonal carcinoma | T1N0M0 | Malignant |
| TE2081 | I6 | M | 38 | Testis | Embryonal carcinoma | T1N0M0 | Malignant |
| TE2081 | I7 | M | 37 | Testis | Embryonal carcinoma | T1N0M0 | Malignant |
| TE2081 | I8 | M | 37 | Testis | Embryonal carcinoma | T1N0M0 | Malignant |
| TE2081 | I9 | M | 17 | Testis | Embryonal carcinoma | T1N0M0 | Malignant |
| TE2081 | I10 | M | 17 | Testis | Embryonal carcinoma (atrophy) | T1N0M0 | Malignant |
| TE2081 | I11 | M | 22 | Testis | Embryonal carcinoma | T2N0M0 | Malignant |
| TE2081 | I12 | M | 22 | Testis | Embryonal carcinoma | T2N0M0 | Malignant |
| TE2081 | I13 | M | 35 | Testis | Immature teratoma | T1N0M0 | Malignant |
| TE2081 | I14 | M | 35 | Testis | Immature teratoma | T1N0M0 | Malignant |
| TE2081 | I15 | M | 32 | Testis | Teratoma with malignant transformation to adenocarcinoma | T1N0M0 | Malignant |
| TE2081 | I16 | M | 32 | Testis | Teratoma with malignant transformation to adenocarcinoma | T1N0M0 | Malignant |
| TE2081 | J1 | M | 44 | Testis | Immature teratoma | T2N0M0 | Malignant |
| TE2081 | J2 | M | 44 | Testis | Immature teratoma | T2N0M0 | Malignant |
| TE2081 | J3 | M | 48 | Testis | Mature teratoma | - | Benign |
| TE2081 | J4 | M | 48 | Testis | Mature teratoma | - | Benign |
| TE2081 | J5 | M | 39 | Testis | Mature teratoma | - | Benign |
| TE2081 | J6 | M | 39 | Testis | Mature teratoma | - | Benign |
| TE2081 | J7 | M | 26 | Testis | Tuberculosis (sparse) | - | Benign |
| TE2081 | J8 | M | 26 | Testis | Tuberculosis (sparse) | - | Benign |
| TE2081 | J9 | M | 40 | Testis | Tuberculosis (sparse) with necrosis | - | Benign |
| TE2081 | J10 | M | 40 | Testis | Tuberculosis (sparse) with necrosis | - | Benign |
| TE2081 | J11 | M | 68 | Testis | Tuberculosis | - | Benign |
| TE2081 | J12 | M | 68 | Testis | Tuberculosis | - | Benign |
| TE2081 | J13 | M | 72 | Testis | Atrophy | - | Benign |
| TE2081 | J14 | M | 72 | Testis | Atrophy | - | Benign |
| TE2081 | J15 | M | 87 | Testis | Atrophy | - | Benign |
| TE2081 | J16 | M | 87 | Testis | Atrophy | - | Benign |
| TE2081 | K1 | M | 61 | Testis | Atrophy | - | Benign |
| TE2081 | K2 | M | 61 | Testis | Atrophy | - | Benign |
| TE2081 | K3 | M | 83 | Testis | Atrophy | - | Benign |
| TE2081 | K4 | M | 83 | Testis | Atrophy | - | Benign |
| TE2081 | K5 | M | 52 | Testis | Atrophy | - | Benign |
| TE2081 | K6 | M | 52 | Testis | Atrophy | - | Benign |
| TE2081 | K7 | M | 68 | Testis | Atrophy | - | Benign |
| TE2081 | K8 | M | 68 | Testis | Atrophy | - | Benign |
| TE2081 | K9 | M | 65 | Testis | Mild atrophy | - | Benign |
| TE2081 | K10 | M | 65 | Testis | Mild atrophy | - | Benign |
| TE2081 | K11 | M | 74 | Testis | Atrophy | - | Benign |
| TE2081 | K12 | M | 74 | Testis | Atrophy | - | Benign |
| TE2081 | K13 | M | 74 | Testis | Cancer adjacent normal testicular tissue | - | NAT |
| TE2081 | K14 | M | 74 | Testis | Cancer adjacent normal testicular tissue | - | NAT |
| TE2081 | K15 | M | 61 | Testis | Cancer adjacent normal testicular tissue | - | NAT |
| TE2081 | K16 | M | 61 | Testis | Cancer adjacent normal testicular tissue | - | NAT |
| TE2081 | L1 | M | 69 | Testis | Cancer adjacent normal testicular tissue | - | NAT |
| TE2081 | L2 | M | 69 | Testis | Cancer adjacent normal testicular tissue | - | NAT |
| TE2081 | L3 | M | 70 | Testis | Cancer adjacent normal testicular tissue | - | NAT |
| TE2081 | L4 | M | 70 | Testis | Cancer adjacent normal testicular tissue | - | NAT |
| TE2081 | L5 | M | 46 | Testis | Cancer adjacent normal testicular tissue | - | NAT |
| TE2081 | L6 | M | 46 | Testis | Cancer adjacent normal testicular tissue | - | NAT |
| TE2081 | L7 | M | 54 | Testis | Cancer adjacent normal testicular tissue | - | NAT |
| TE2081 | L8 | M | 54 | Testis | Cancer adjacent normal testicular tissue | - | NAT |
| TE2081 | L9 | M | 35 | Testis | Cancer adjacent normal testicular tissue | - | NAT |
| TE2081 | L10 | M | 35 | Testis | Cancer adjacent normal testicular tissue | - | NAT |
| TE2081 | L11 | M | 48 | Testis | Cancer adjacent normal testicular tissue | - | NAT |
| TE2081 | L12 | M | 48 | Testis | Cancer adjacent normal testicular tissue | - | NAT |
| TE2081 | L13 | M | 21 | Testis | Cancer adjacent normal testicular tissue | - | NAT |
| TE2081 | L14 | M | 21 | Testis | Cancer adjacent normal testicular tissue | - | NAT |
| TE2081 | L15 | M | 39 | Testis | Cancer adjacent normal testicular tissue | - | NAT |
| TE2081 | L16 | M | 39 | Testis | Cancer adjacent normal testicular tissue | - | NAT |
| TE2081 | M1 | M | 56 | Testis | Cancer adjacent normal testicular tissue | - | NAT |
| TE2081 | M2 | M | 56 | Testis | Cancer adjacent normal testicular tissue | - | NAT |
| TE2081 | M3 | M | 57 | Testis | Cancer adjacent normal testicular tissue | - | NAT |
| TE2081 | M4 | M | 57 | Testis | Cancer adjacent normal testicular tissue | - | NAT |
| TE2081 | M5 | M | 69 | Testis | Cancer adjacent normal testicular tissue | - | NAT |
| TE2081 | M6 | M | 69 | Testis | Cancer adjacent normal testicular tissue | - | NAT |
| TE2081 | M7 | M | 28 | Testis | Normal testicular tissue | - | Normal |
| TE2081 | M8 | M | 28 | Testis | Normal testicular tissue | - | Normal |
| TE2081 | M9 | M | 30 | Testis | Normal testicular tissue | - | Normal |
| TE2081 | M10 | M | 30 | Testis | Normal testicular tissue | - | Normal |
| TE2081 | M11 | M | 45 | Testis | Normal testicular tissue | - | Normal |
| TE2081 | M12 | M | 45 | Testis | Normal testicular tissue | - | Normal |
| TE2081 | M13 | M | 45 | Testis | Normal testicular tissue | - | Normal |
| TE2081 | M14 | M | 45 | Testis | Normal testicular tissue | - | Normal |
| TE2081 | M15 | M | 46 | Testis | Normal testicular tissue | - | Normal |
| TE2081 | M16 | M | 46 | Testis | Normal testicular tissue | - | Normal |

*Remark: detailed pathology report can be obtained online: http://www.biomax.us/tissue-arrays/Testis/*
